# Supplementary material for: Angiogenesis in the Outer Membrane of Chronic Subdural Hematomas through Thrombin-Cleaved Osteopontin and the Integrin α9 and Integrin β1 Signaling Pathways
Source: Biomedicines. 2023 May 13;11(5):1440. doi: 10.3390/biomedicines11051440 (PMC10216439; doi:10.3390/biomedicines11051440)
Supplement: Supplementary file 1 [file biomedicines-11-01440-s001.zip › biomedicines-2389423-supplementary.pdf]

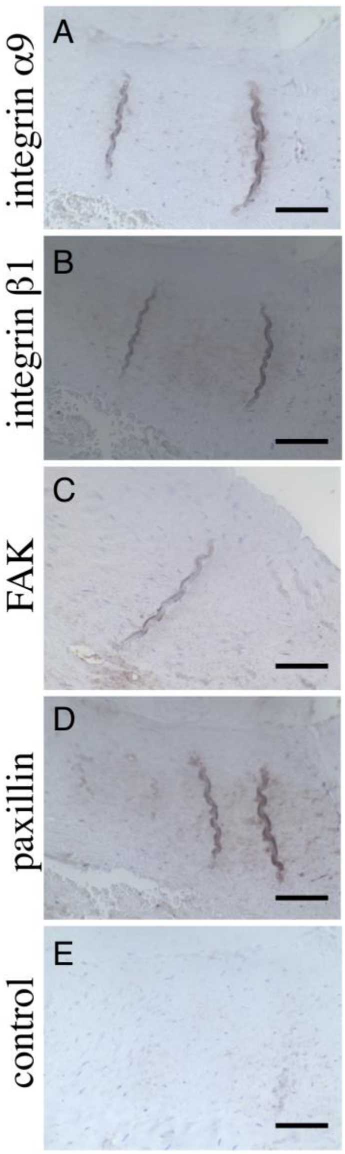

Supplemental Figure S1. Immunohistochemical analysis of dura mater adjacent to chronic subdural hematoma membranes. Ten-micrometer consecutive slices were immunostained with polyclonal antibodies against integrin α9 (A), integrin β1 (B), focal adhesion kinase (FAK, C) and paxillin (D) using the ABC method. Note that there was no significant staining of these molecules except the endothelial cells in small tortuous arteries penetrating the dura mater. Slices immunostained without primary antibodies are shown in (E). Scale bars = 100 μm.
